# Surgical treatment of double primary liver cancer: An observational study for a rare type of tumor

**DOI:** 10.1097/MD.0000000000004412

**Published:** 2016-08-12

**Authors:** Aijun Li, Senlin Ma, Timothy Pawlik, Bin Wu, Xiaoyu Yang, Longjiu Cui, Mengchao Wu

**Affiliations:** a Department of Second Special Treatment, Eastern Hepatobiliary Surgery Hospital, the Second Military Medical University, Shanghai, China; b Department of Surgery, Johns Hopkins Hospital, Baltimore, MD, USA.

**Keywords:** clinical characteristics, double primary liver cancer, prognosis, surgical treatment

## Abstract

Double primary liver cancer (DPLC) is a special type of clinical situation. As such, a detailed analysis of the surgical management and prognosis of patients with DPLC is lacking. The objective of the current study was to define the management and outcome of patients undergoing surgery for DPLC at a major hepatobiliary center.

A total of 87 patients treated by surgical resection at the Eastern Hepatobiliary Surgery Hospital from January 1st, 2007 to October 31st, 2013 who had DPLC demonstrated by final pathological diagnosis were identified. Among these, 50 patients had complete clinical and prognostic data. Demographic and tumor characteristics as well as the prognosis were analyzed.

The proportion of hepatitis B surface antigen (HBsAg) (+) and hepatitis B virus e antigen (HBeAg) (+), HBsAg (+), and HBeAg (−) hepatocirrhosis in all patients was 21.84%, 67.82%, and 63.22%, respectively. Incidental findings accounted for 58.62% of patients; among those who had symptoms, the main symptom was abdominal pain (31.03%). Nonanatomic wedge resection was the main operative approach (62.07%). Postoperatively, the main complications included seroperitoneum (11.49%), hypoproteinemia (10.34%), and pleural effusion (8.05%). Factors associated with disease-free survival (DFS) included intrahepatic cholangiocarcinoma (ICC) tumor size (*P* = 0.002) and use of postoperative prophylactic transcatheter arterial chemoembolization (TACE) treatment (*P* = 0.015). Meanwhile, hepatocellular carcinoma (HCC) size (*P* = 0.045), ICC size (*P* < 0.001), and liver function (including aspartate aminotransferase [*P* = 0.001] and r-glutamyl transferase [*P* < 0.001]) were associated with overall survival (OS).

Hepatitis B virus (HBV)-related hepatitis or cirrhosis is also an important factor in the pathogenesis of DPLC and surgical treatment is safe for it with low complication rates. In addition, it is effective to prolong DFS that DPLC patients undergo postoperative prophylactic TACE treatment.

## 1 Introduction

Primary carcinoma of liver is one of the most common malignant tumors worldwide. Primary tumors of the liver can typically be divided into 3 types according to World Health Organization ^[1]^: hepatocellular carcinoma (HCC), intrahepatic cholangiocarcinoma (ICC), and combined hepatocellular and cholangiocarcinoma. Combined hepatocellular and cholangiocarcinoma, which account for 0.54% to 14.2% of all primary liver cancers, [^2^ ^3^] can be subsequently divided into 3 subtypes according to Allen and Lisa ^[4]^: type A, HCC and ICC exist in 2 independent and simultaneous masses in a liver without any connection between the masses (double primary liver cancer [DPLC]); type B, 2 consecutive but independent masses contain HCC and ICC (combined type); type C, 1 isolated mass contains HCC and ICC simultaneously (double cancer).

The incidence of DPLC is very low, with 37 total cases reported in the literature worldwide. [^5^ ^6^ ^7^ ^8^ ^9^ ^10^ ^11^ ^12^ ^13^ ^14^] Previous research has demonstrated an association between hepatitis C virus (HCV) infection and generation or development of DPLC. ^[8]^ Once present, the main treatment of DPLC is surgical resection. However, only a few case reports have been published due to the very low incidence of DPLC. In one case report, Watanabe et al ^[8]^ examined the correlation of size, location, and liver cirrhosis with DPLC. A detailed analysis of the surgery management and prognosis of patients undergoing surgery for DPLC is still lacking. As such, data on this rare malignant tumor are important as such information may provide greater insight into surgical management of DPLC and inform future treatment strategies.

The present study investigated the management and outcomes of 87 patients with DPLC documented on final surgical pathology. In addition, the demographic, tumor-specific factors, and operative outcomes, including prognosis, of DPLC patients who underwent surgical resection were defined.

## 2 Methods

### 2.1 Patients selection

The present study was approved by Committee on Ethics of the Eastern Hepatobiliary Surgery Hospital, Second Military Medical University. Besides, informed consents that included the purpose and methods of the study as well as the using of hospitalization information and the tumor specimens of patients were given to every DPLC patient or their immediate family members of this study. And obtained the consents of every DPLC patient or their immediate family members. A total of 87 patients treated by surgical resection at Eastern Hepatobiliary Surgery Hospital from January 1st, 2007 to October 31st, 2013 who had documented DPLC on final pathological analysis were included in this study. Inclusion and exclusion criteria included: computed tomography (CT) or magnetic resonance imaging (MRI) together with liver and kidney function; no other treatment except embolization was performed before operation. Surgery consisted of resection, enucleation, ablation and One alinjection. Types of hepatic resection included nonanatomic wedge resection, segmentectomy, bisegmentectomy, right hepatectomy (segments V, VI, VII, and VIII), left hepatectomy (segments II, III, and IV), extended right hepatectomy (segments IV, V, VI, VII, and VIII), extended left hepatectomy (segments II, III, IV, V, and VIII), central hepatectomy (segments IV, V, and VIII), and liver transplant according to the liver segments dividing method of Strasberg. ^[15]^

The postoperative treatment is prophylactic transcatheter arterial chemoembolization (TACE). Prophylactic TACE treatment is defined as: patients came to our hospital or the local hospital voluntarily within 2 months after primary resection with good liver and kidney function, no obvious taboo, as well as no obvious disorder blood vessels and tumor staining during TACE.

### 2.2 Data collection

The basic data of patients include gender and age. DPLC characteristics included intrahepatic distribution, location, and tumor size of HCC and ICC. Preoperative liver function included the serum levels of albumin, total bilirubin, and international normalized ratio and the course of cirrhosis, etc. The history of alcoholism was also collected and the standard of alcoholism is as follows: the time of drinking >5 years, daily alcohol intake >80 g. Ethanol (g) = Alcohol Consumption (mL) × Ethanol Concentration (%) × 0.8. ^[16]^ Clinical manifestations included incidental findings, abdominal pain/discomfort, nausea/vomiting, fatigue, and rupture. Data on operative characteristics including surgical indications, methods of surgical treatment, resection range, blood loss and transfusion, and pathological results were also collected. Postoperative complications included bile leakage, bleeding, liver failure, renal failure, etc. Postoperative mortality included 2 time points: 30 and 90 days after surgery.

### 2.3 Immunohistochemistry

Representative 4-μm serial sections were prepared from 10% formalin-fixed, paraffin-embedded tissue blocks. Deparaffinization was conducted by treating the cells with xylene for 10 minutes; 100% ethanol for 1 minutes, 95% ethanol for 1 minutes, 85% ethanol for 1 minutes, and 75% ethanol for 1 minutes. Briefly, all slides were exposed to 3% hydrogen peroxide for 10 minutes to block endogenous peroxidase activity. Microwave antigen retrieval was performed in citrate buffer (pH 6.0) for 5 minutes to increase the immunoreactivity. Microwaving at medium/high heat for 15 minutes, keeping the solution boiling, followed by treatment with 5% skimmed milk in phosphate-buffered saline–0.1% bovine serum albumin for a minimum of 1 hour at room temperature to block nonspecific staining.

Hep Par 1 (cytoplasm of hepatocytes, 1:50, Dako) and CK-19 (cytoplasm of cholangiocytes, 1:50, Dako) were added to HCC and ICC tissue, respectively. Sections were incubated with primary antibodies in a humid chamber at 4 °C overnight, followed by incubation with antimouse peroxidase-conjugated envision antibody at 37 °C for 30 minutes. Immunoreactions were visualized by 3,3-diaminobenzidine as chromogen for 5 minutes at room temperature, followed by light counterstaining with hematoxylin. Tissue structures were visualized by counterstaining with hematoxylin (Bioengineering, Shanghai, Ltd., China).

### 2.4 Follow-up

**Figure 1:**
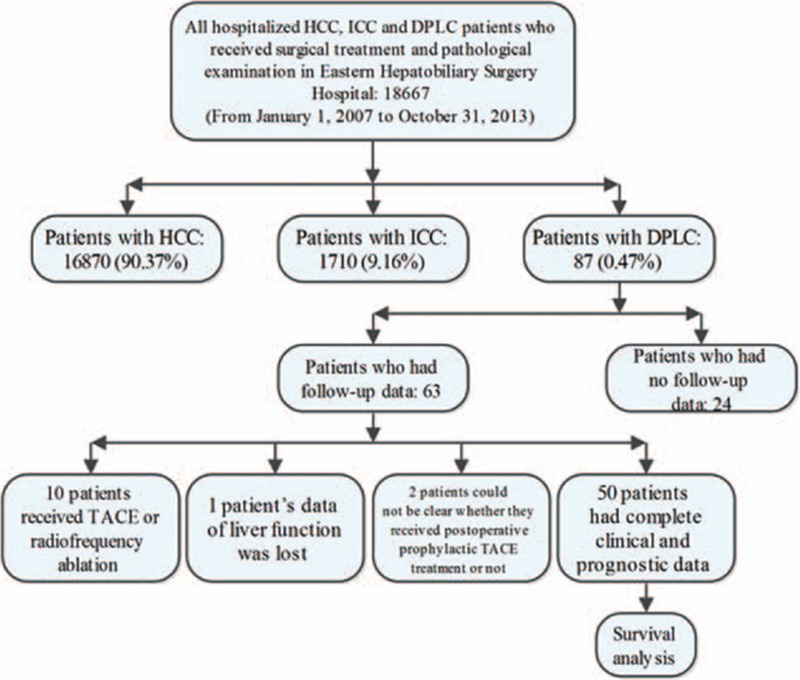
The flowchart of patients’ collection in this study.

In order to make sure that each patient was followed for no less than 2 years, the last date of follow-up for any patient in this study was November 1, 2015. Follow-up data including recurrence/recurrence time and death/death time of 63 patients among the total 87 patients were obtained. Ten patients among the 63 patients were treated with TACE and (or) radiofrequency ablation before operative. Among the remaining 53 patients, 1 patient's liver function data were lost and 2 patients’ prophylactic TACE treatment were not clearly defined. The number of patients who had complete clinical and prognostic data was 50. The disease-free survival (DFS) time and the overall survival (OS) time of these 50 cases were analyzed by life tables. At the same time, the influencing factors of DFS and OS were analyzed by Cox regression analysis. The flowchart of the above patients’ collection in this study was given in Fig. 1.

### 2.5 Statistical analysis

Continuous variables were represented by mean (±standard deviation) or median values (interquartile range), respectively, according to the characteristics of the data and whether the data were normal distribution. Discrete variables were represented by total + frequency. The independent sample *t* test or rank test was used to compare 2 groups of continuous variables. DFS and OS of the 50 patients who had complete outcome data were analyzed using life tables and the survival curves compared with Kaplan–Meier analyses. The influencing factors of DFS and OS were analyzed by Cox regression with the method of “ENTER.” Statistical significance was set at α = 0.05. SPSS18.0 (Institute Inc IBM, New York) was used for statistical analysis of all data.

## 3 Results

The number of DPLC patients who received surgical treatment was 87 and demographics and tumors characteristics were shown in Table 1. The median age (interquartile range) was 52.46 (45–61). There were a total of 78 male patients and male:female ratio was 8.67:1. There was no significant statistical difference between the volume (cm) of HCC and ICC (*P* = 0.610, 2 independent sample *t* test) present in the liver. HCC multiple cases and ICC multiple cases were found in the present study. There were 6 patients (6.90%) who had multiple HCC and 3 patients (3.45%) who had multiple ICC. There was 1 patient with multiple ICC and multiple HCC at the same time and the HCC diameter was 7 cm + 4 cm, meanwhile the ICCs’ was 2.4 cm + 1.4 cm. In the aspects of tumors’ location: the most common was locating on the right lobe synchronously (44 patients, 50.57%) and the rest of the locations were rare. More details were given in Table 1. The proportion of hepatitis B surface antigen (HBsAg) (+) and hepatitis B virus e antigen (HBeAg) (+), HBsAg (+), and HBeAg (−) hepatocirrhosis in all patients was 21.84%, 67.82%, and 63.22%, respectively. There was only 1 patient with no hepatitis B virus (HBV) and HCV infection; however, moderate hepatic adipose infiltration and chronic cholecystitis were found in postoperative pathological examination. There was also 1 patient with both HBV and HCV infection. In addition, there were 10 patients had blood carbohydrate antigen (CA) −242 levels assessed, among whom 9 (90.00%) patients had a slightly elevated value. In terms of clinical manifestation, incidental findings accounted for 58.62% with the main symptom of the disease was abdominal pain (31.03%).

**Table 1:**
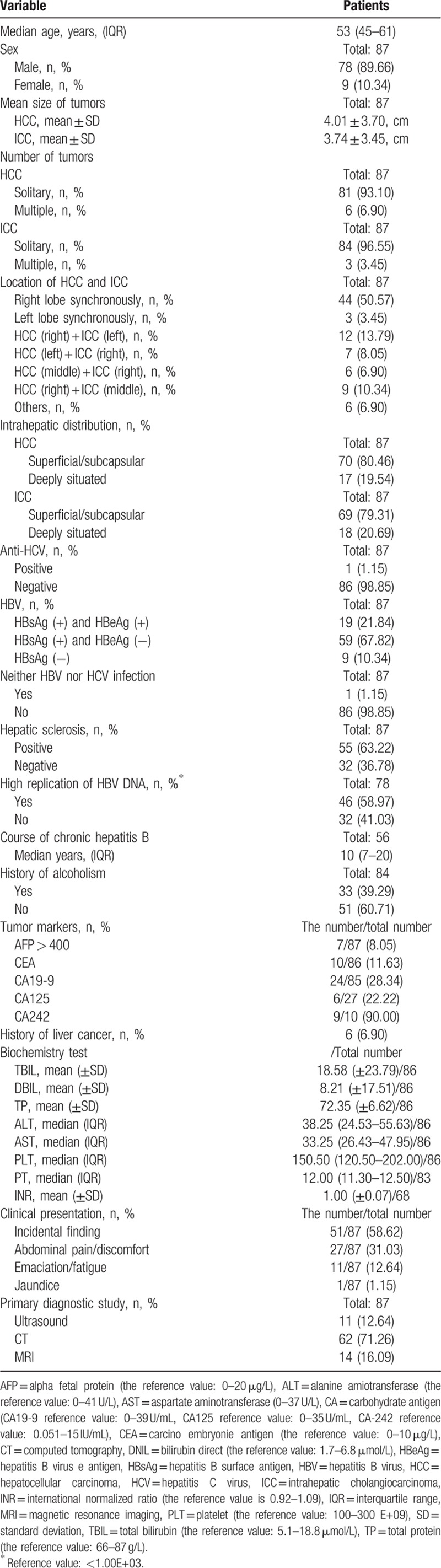
Demographics and tumors characteristics in patients that underwent liver resection.

Operative details and perioperative outcomes were noted in Tables 2 and 3. Nonanatomic wedge resection was the main operative approach (62.07%) as most tumors were located in the peripheral segment of the liver. Among the 63 patients who had complete follow-up data, there was 1 patient (1.59%) who was readmitted within 30-days and was managed conservatively for a severe biliary leakage after surgery; there was 1 patient (1.59%) who died within 30 days after surgery because of respiratory failure. In addition, 1 patient experienced short-term recurrence and died on the 60th day after operation.

**Table 2:**
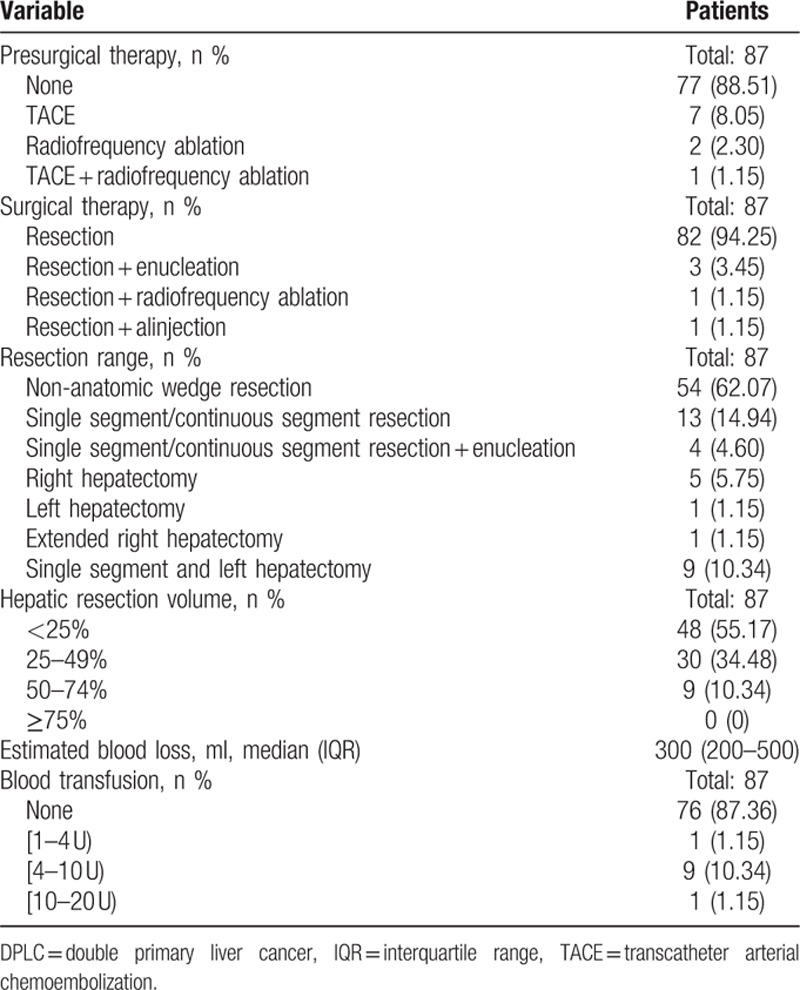
Operative details of patients that underwent DPLC resection.

**Table 3:**
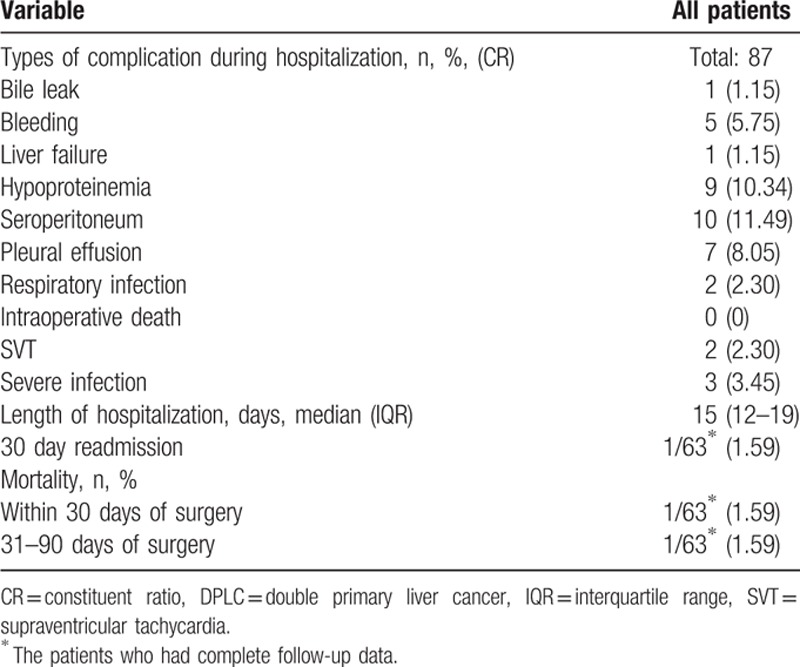
Perioperative outcomes following DPLC resection.

**Figure 2:**
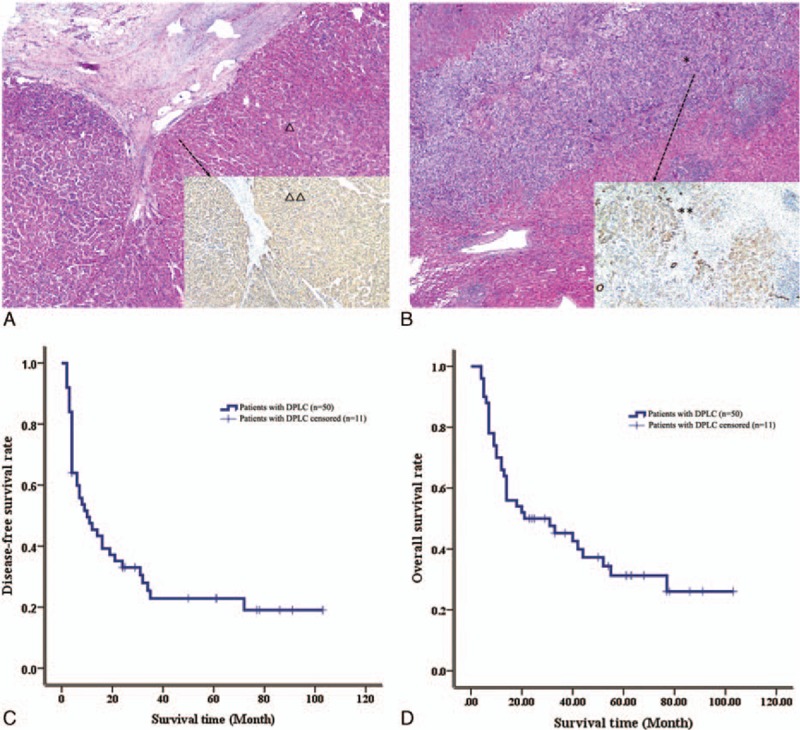
The postoperative pathological examination and survival of patients with DPLC. (A) The HCC in DPLC (△) and Hep Par 1 was asystematic positive in HCC (△△). (B) The ICC in DPLC (∗) and CK-19 was local positive in ICC (∗∗). (C) The DFS rate of patients with DPLC. (D) The overall survival rate of patients with DPLC. HCC = hepatocellular carcinoma, DFS = disease-free survival, DPLC = double primary liver cancer, ICC = intrahepatic cholangiocarcinoma.

HE results from the 2 simultaneous masses obtained from the same patient proved that the 2 tumors were pure HCC and ICC, respectively. Hep Par 1 was asystematic positive in HCC and CK-19 was local positive in ICC. The results of immunohistochemistry confirmed the results of HE staining. HE and immunohistochemical staining of were performed in DPLC specimens of 87 patients and the typical pathological results were shown in Fig. 2A and B.

Survival analysis of the 50 patients who had complete clinical and follow-up data with no preoperative treatment was shown in Table 4. Median DFS ± standard error (SE) was 10.00 ± 4.02 (month) (95% confidence interval was 2.11–17.89) (Fig. 2C); recurrence was noted in 23 patients within an average of 9 months’ time for a recurrence rate of 46.00%. There were 38 patients with recurrence (76.00%) and 12 patients with no recurrence (24.00%) in all of the 50 patients with complete follow-up data. The detailed sites of recurrence which were diagnosed for the first time in 38 patients were as follows – intrahepatic recurrence: 33 patients (86.84%); intrahepatic recurrence + retroperitoneal lymph node metastasis: 2 patients (5.26%); portal vein tumor thrombus 1 patients (2.63%); tumor metastasis in the abdominal wall under xiphoid: 1 patients (2.63%); intrahepatic recurrence + right femoral intertrochanteric metastasis: 1 patients (2.63%). Median OS ± SE = 21.00 ± 13.70 (month) (95% confidence interval was 2.11–17.89) (Fig. 2D). There were no deaths within the first 3 months among the 50 patients analyzed. In contrast, a total of 22 patients died from the 3rd to 14th months after the operation for a mortality of 44%. There were 33 patients died (66.00%) and 17 patients who were still alive (34.00%) in all of the 50 patients with complete follow-up data before the end of the following up. And the death reasons of the 33 dead patients were as follows: 32 patients (96.97%) died from cancer and 1 patient (3.03%) died from iliac passion caused by some other reason. Remarkably, there was 1 patient who underwent an operation on February 14, 2007 who survived for 103 months with no recurrence and metastasis at the time of last follow-up. In addition, there were 3 patients with no recurrence and metastasis with a survival time of 86, 78, and 77 months, respectively.

**Table 4:**
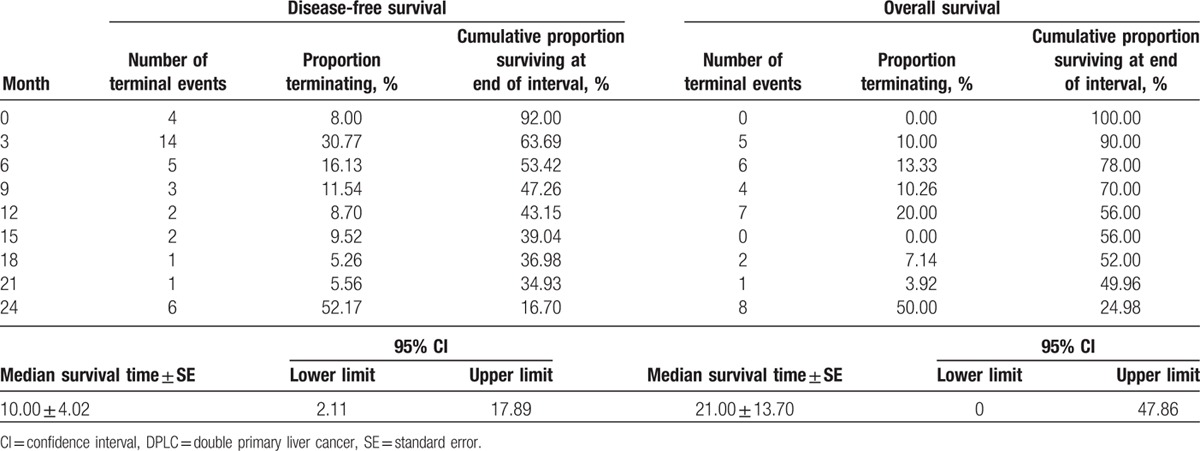
Survival analysis of patients with DPLC after surgical resection.

Cox regression analysis of prognostic factors associated with outcomes for these 50 DPLC cases is presented in Table 5. Factors associated with DFS included ICC tumor size (*P* = 0.002) and use of postoperative prophylactic TACE treatment (*P* = 0.015). Meanwhile, HCC size (*P* = 0.045), ICC size (*P* < 0.001), and liver function (including aspartate aminotransferase [*P* = 0.001] and r-glutamyl transferase [*P* < 0.001]) were associated with OS.

**Table 5:**
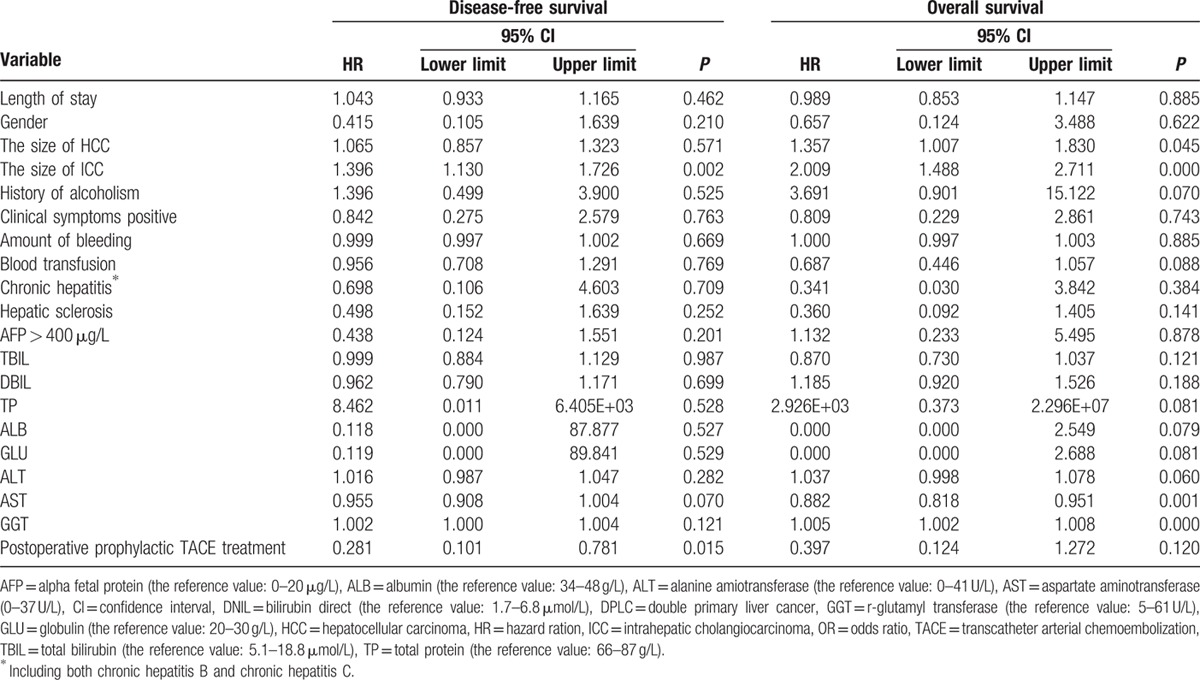
Cox regression analysis of prognostic factors for 50 cases DPLC with complete data.

## 4 Discussion

DPLC is a rare type of liver tumor in which HCC and ICC exist as 2 independent and simultaneous masses in 1 liver with no common connection between the masses. ^[4]^ Data on management and outcomes of patients with DPLC are scarce due to its extremely low incidence. Demographic and tumor characteristics as well as the prognosis were analyzed in the present study provide a detailed account of the diagnosis, treatment, and outcome of patients diagnosed with DPLC.

Previous reports confirmed that HCV-associated viral hepatitis or cirrhosis may play an important role in pathogenesis of DPLC[ ^7^ ^8^] while DPLC with HBV infection is rare. ^[9]^ For example, the report of 33 DPLC cases around the world by Watanabe et al ^[8]^ confirmed that 72.7% (24 cases) of patients were anti-HCV positive, while only 9.3% (3 cases) of patients were antigen HBs positive. This study found, however, that the proportions of HBsAg (+) and HBeAg (+), HBsAg (+) and HBeAg (−) in all 87 patients were 21.84%, 67.82%, respectively. The anti-HCV positive cases accounted for only 1.15%. These data were different from the previous studies. It is well-established, however, that hepatitis or liver cirrhosis caused by HBV or HCV infection is an important factor associated with the risk of HCC or ICC. [^17^ ^18^ ^19^] We believe that HBV-related hepatitis or cirrhosis is also an important factor in the pathogenesis of DPLC based on the results of the present and previous studies. The reason why HBV and HCV infection rates were different in various studies may be the regional differences of hepatitis viruses’ types. For example, hepatitis virus types in China is mainly HBV and the role of HBV infection in DPLC pathogenesis is more significant. ^[9]^

The pathogenesis of DPLC as related to the hepatic progenitor cell (HPC) was found to play an important role in tumor carcinogenesis reported in recent years. HPC which can differentiate into hepatic cells as well as bile duct cells, as it is a kind of progenitor cell with bi directional differentiation potential.[ ^20^ ^21^] HPC with malignant transformation under the action of some “initiator” (including chronic inflammation) also has the same potential. DPLC may occur after a period of proliferation and differentiation of HPC with malignant transformation. ^[8]^ On the other hand, the mature hepatocytes and bile duct cells may also be affected by chronic inflammation and other factors and the 2 types of cells may proliferate with malignant transformation at the same time. In turn, DPLC may then form eventually.

Interestingly, there was 1 patient who has neither HBV nor HCV infection. Then we reviewed the medical history. We found that the patient was diagnosed as right HCC and was treated with right liver tumor resection before 18 months when DPLC was diagnosed. This patient was diagnosed with thyroid cancer in 1999 and was treated with concurrent resection. In addition, moderate hepatic adipose infiltration and chronic cholecystitis were found in postoperative pathological examination of DPLC resection. Therefore, it is worth further study whether liver or other systemic diseases including hepatic adipose infiltration can affect the tumorigenesis of DPLC.

Preoperative diagnosis of DPLC is more difficult at present due to its low incidence and the lack of awareness of clinicians. Its diagnosis mainly depends on pathological examination. The preoperative CT or MRI always make “atypical liver cancer” as a primary diagnosis and cannot give an accurate diagnosis in many cases. ^[8]^ The primary masses (or the larger masses) features of enhanced CT or MRI image are “fast wash-in and fast wash-out,” which are the main features of HCC, or “delayed reinforcement,” which are the main features of ICC. So, clinicians can make a diagnosis of “liver malignant tumor (tend to be HCC or ICC)” for DPLC patients. But the secondary masses (or the smaller masses) were always considered to be the satellite nodules or intrahepatic metastasis of primary tumors. Some smaller lumps could not be found in CT or MRI before surgery and they were discovered accidentally during surgery. In fact, the CT or MRI images of DPLC are mainly the combinations of the image features with HCC and ICC. Clinicians should consider the possibility of DPLC even if its incidence is very low when the same image is characterized by HCC and ICC. In addition, alpha fetal protein, CA19-9, and other tumor markers were not sensitive and specific enough to diagnose DPLC. For example, there were 10 patients who had a detected blood CA-242 level but the level was only slightly elevated in 9. So, this kind of tumor markers can only provide reference in differentiation of benign and malignant tumors and no exact diagnosis value for DPLC. It is worth noting that they should be treated actively once the “liver malignant tumor” was diagnosed.

The patients can be treated with appropriate TACE or ablation before operation according to the individual differences. Other flexible surgical methods (including enucleation, ablation, or anhydrous alcohol injection mentioned in the present study) can be considered according to the size and location of the tumors. Surgical treatment is safe. Specifically, there were no death cases in hospital of all 87 DPLC patients. Seroperitoneum (11.49%), hypoproteinemia (10.34%), and pleural effusion (8.05%) were the mainly postoperative complications. These complications may have been prevented with protein complement and diuresis actively after the operation.

Most importantly, a detailed statistical analysis of both postoperative survival and the factors associated with outcome among 50 patients with DPLC was performed in the present study. The median DFS time (median ± SE) of 50 patients was 10.00 ± 4.02 (month), and the median OS time (median ± SE) was 21.00 ± 13.70 (month). The highest postoperative recurrence rate was the in 1st 9 months at 46.00%; recurrence was actually highest from 3 to 6 months with 17 patients experience a recurrence during this time period. Then the rate declined gradually after 9th months. In addition, the postoperative mortality of 0 to 3rd months (not including the 3rd month) was low as only 1 patient dying of respiratory failure. Then the rate then increased gradually and the highest mortality rate was in 3rd to 12th months (not including the 12th month) after operation with 44.00%. The postoperative mortality showed an abrupt decreasing after 12 months. From the above data we can know that the 1st year after operation may the high-risk period for recurrence and death in patients with DPLC. Reexamining after surgery as well as active treatment of complications and recurrence may reduce the recurrence and mortality rates of DPLC patients effectively during this period.

In terms of DFS and OS Cox, we noted that the ICC tumor size can affect not only patient's DFS (*P* = 0.002), but also OS (*P* < 0.001), while HCC tumor size can only effect OS (*P* < 0.045). We also noticed that such patients relapse in form of lung metastasis in the terminal stage of cancer, which is consistent with the biological behavior of ICC. In turn, we speculated that the influence of ICC on DFS, OS, or even the overall situation of patients with DPLC was greater than the influence of HCC. As such, for patients with DPLC, the clinician may need to pay more attention to the development and management of the ICC as compared with HCC. In addition, it is worth noting that postoperative prophylactic TACE treatment is a protective factor for DFS (*P* = 0.015), even though it has no effect on OS of patients with DPLC. So, it is effective to prolong DFS that DPLC patients undergo postoperative prophylactic TACE treatment. Therefore, we recommend that the patients can receive postoperative prophylactic TACE treatment within 1 to 2 months after resection of DPLC if they have no exact contraindication in order to decrease the recurrence and prolong survival for DPLC patients.

In conclusion, we have carried on a detailed analysis of the diagnosis and treatment of the 87 patients with DPLC and share the experience in this study. Surgical treatment is safe for the patients of DPLC. The 1st choice of treatment for patients with DPLC is curative resection according to previous reports and the results of this study, ^[8]^ and other flexible surgical methods (including enucleation, ablation, or anhydrous alcohol injection mentioned in the present study) can be considered according to the size and location of the tumors. Besides, postoperative prophylactic TACE treatment can be recommended to decrease the recurrence and prolong survival for DPLC patients and the serious complications mentioned in this study should be paid more attention. It needs further study to research whether other treatment options (radiotherapy and targeted drug therapy) are effective and more consensus still needs further research.

Although the current study has several limitation and was limited due to sample size, it does provide the largest report of patients with DPLC managed with resection to date. In our future study, we may make comparative analysis of the infection characteristics, surgical outcomes, and other aspects of DPLC with other liver tumor (including pure HCC or ICC), and summarize the characteristics of DPLC in the aspect of clinical diagnosis and treatment. Second, this study was only limited to the clinical analysis and lack of in-depth study for the pathogenesis of DPLC. In the further study, we may make fundamental research (including genetic testing) on the frozen tumor tissues of the typical DPLC patients to find coexpressed or differential expressed genes. Making comparison between DPLC and pure HCC or ICC might help us explore the pathogenesis and key genes of this 3 kind of tumors, as well as the differences and relations with each other. The follow-up study will be remarkable.
